# Myotubes from Severely Obese Type 2 Diabetic Subjects Accumulate Less Lipids and Show Higher Lipolytic Rate than Myotubes from Severely Obese Non-Diabetic Subjects

**DOI:** 10.1371/journal.pone.0119556

**Published:** 2015-03-19

**Authors:** Siril S. Bakke, Yuan Z. Feng, Natasa Nikolić, Eili T. Kase, Cedric Moro, Camilla Stensrud, Lisbeth Damlien, Marianne O. Ludahl, Rune Sandbu, Brita Marie Solheim, Arild C. Rustan, Jøran Hjelmesæth, G. Hege Thoresen, Vigdis Aas

**Affiliations:** 1 Department of Pharmaceutical Biosciences, School of Pharmacy, University of Oslo, Oslo, Norway; 2 Institut National de la Santé et de la Recherche Médicale, Unité Mixte de Recherche 1048, Obesity Research Laboratory, Institute of Metabolic and Cardiovascular Diseases, University of Toulouse, Toulouse, France; 3 Faculty of Health, Oslo and Akershus University College of Applied Sciences, Oslo, Norway; 4 The Morbid Obesity Center, Vestfold Hospital Trust, Tønsberg, Norway; 5 Department of Pharmacology, Institute of Clinical Medicine, Faculty of Medicine, University of Oslo and Oslo University Hospital, Oslo, Norway; Università “Magna Graecia” di Catanzaro, ITALY

## Abstract

About 80% of patients with type 2 diabetes are classified as overweight. However, only about 1/3 of severely obese subjects have type 2 diabetes. This indicates that several severely obese individuals may possess certain characteristics that protect them against type 2 diabetes. We therefore hypothesized that this apparent paradox could be related to fundamental differences in skeletal muscle lipid handling. Energy metabolism and metabolic flexibility were examined in human myotubes derived from severely obese subjects without (BMI 44±7 kg/m^2^) and with type 2 diabetes (BMI 43±6 kg/m^2^). Lower insulin sensitivity was observed in myotubes from severely obese subjects with type 2 diabetes. Lipolysis rate was higher, and oleic acid accumulation, triacylglycerol content, and fatty acid adaptability were lower in myotubes from severely obese subjects with type 2 diabetes compared to severely obese non-diabetic subjects. There were no differences in lipid distribution and mRNA and protein expression of the lipases HSL and ATGL, the lipase cofactor CGI-58, or the lipid droplet proteins PLIN2 and PLIN3. Glucose and oleic acid oxidation were also similar in cells from the two groups. In conclusion, myotubes established from severely obese donors with established type 2 diabetes had lower ability for lipid accumulation and higher lipolysis rate than myotubes from severely obese donors without diabetes. This indicates that a difference in intramyocellular lipid turnover might be fundamental in evolving type 2 diabetes.

## Introduction

Overweight and obesity are strongly associated with insulin resistance and type 2 diabetes, and the majority of subjects with type 2 diabetes are classified as overweight or obese [1]. However, a study at the Morbid Obesity Center in Norway revealed that only 31% of the severely obese patients enrolled for screening (BMI > 35 kg/m^2^) had type 2 diabetes [2]. Many organs are involved in obesity-related type 2 diabetes, but skeletal muscle is one of the organs where insulin resistance is most prominent.

Skeletal muscle uses both fat and carbohydrate as fuel, and fat predominates during fasting. Metabolic flexibility is defined as the muscle’s ability to change between predominantly fatty acid oxidation in the fasting state and carbohydrate oxidation in the fed state (reviewed in [3]). This flexibility is reduced in insulin resistance and type 2 diabetes *in vivo* [4]. Treatments that have positive effects on muscle metabolism, such as omega-3 fatty acids, have been observed to increase both lipid accumulation and metabolic flexibility [5]. Interestingly, it has been observed that muscle cells isolated from overweight/obese patients with or without type 2 diabetes maintain these characteristics when grown in culture [6–8]. Studies performed on cultured skeletal muscle cells (myotubes) from severely obese subjects are few [9–13], but the main findings from these studies are that myotubes from the severely obese subjects have a reduced complete fatty acid oxidation compared to cells from lean subjects [10, 11, 13], in addition to a reduced mitochondrial content [11]. Other studies on myotubes from obese subjects with diabetes (BMI≥30 kg/m^2^) have shown reduced lipid oxidation associated with obesity and type 2 diabetes [14–16]. This reduced oxidation in obese/diabetic muscle has been attributed to impaired mitochondrial capacity [17] or lower mitochondrial content [18]. There are also reports on unaltered and/or increased fatty acid oxidation in human skeletal muscle of obese or insulin resistant individuals [19–21]. Increased fatty acid uptake [11] and partitioning of lipids towards storage rather than oxidation was observed in myotubes from severely obese donors compared to lean donors [10, 11]. Skeletal muscle store fat as triacylglycerols (TAG) in lipid droplets (LDs) and lipid droplet-binding proteins (perilipins) coat and regulate lipid droplet biogenesis and turnover, while adipose triglyceride lipase (ATGL) and hormone sensitive lipase (HSL) are enzymes involved in lipolysis [22–24]. Increased intramyocellular lipid storage and/or levels of intermediates in fatty acid metabolism have been shown to correlate with decreased insulin sensitivity (reviewed in [25]). However, insulin signaling was also improved in presence of increased lipid accumulation in human myotubes [26], and treatment of myotubes with metformin has been shown to increase lipid accumulation in cells from lean individuals [8]. Finally, athletes that are highly insulin sensitive have a higher content of lipid in skeletal muscle than both overweight sedentary and overweight type 2 diabetic individuals [27]. Based on these findings, new theories have emerged that focus on abnormal lipid storage or TAG lipolysis rather than lipid storage *per se* (reviewed in [25]).

Myotubes in culture are known to maintain many phenotypic characteristics of the donor. We wanted to study lipid storage and turnover capacity, as well as oxidation and metabolic flexibility of myotubes from severely obese with and without type 2 diabetes, to see whether lipid handling is inherently different between those who develop type 2 diabetes and those who don’t.

## Materials and Methods

### Materials

Dulbecco’s modified Eagles medium (DMEM-Glutamax) low glucose with sodium pyruvate, DMEM without phenol red, heat-inactivated FCS, penicillin-streptomycin and amphotericin B were from Gibco Invitrogen (Gibco, Life Technologies, Paisley, UK). SkGM-bulletkit was from Lonza (Wakersville, MD, USA). Ultroser G was from PALL (St-Germain-en-Laye, France), insulin (Actrapid) from NovoNordisk (Bagsvaerd, Denmark), BSA, L-carnitine, oleic acid (OA, 18:1, n-9), and triacsin C from Sigma (St.Louis, MO, US). [1–^14^C]oleic acid (2.15 GBq), [9,10-^3^H]triolein (37 MBq) and D-[^14^C-(U)]glucose (107 MBq or 185 MBq) were from PerkinElmer NEN (Boston, MA, US), 2-[^3^H] deoxy-D-glucose (370 GBq) from American Radiolabeled Chemicals Inc. (St.Louis, MO, US). 96-well Scintiplate was from Perkin Elmer (Boston, MA, US), Corning CellBIND tissue culture plates and flasks from Corning Life-Sciences (Schiphol-Rijk, The Netherlands), glass bottom 6-well plates from MatTek (Ashland, MA, US), and Biocoat 25cm^2^ cell flask from BD Biosciences (Franklin Lakes, NJ, US). MitoTrackerRed FM, Hoechst 33258, primers for TaqMan qPCR from Molecular Probes, Invitrogen (Carlsbad, CA, US). RNeasy minikit was from Qiagen (Venlo, The Netherlands), SYBR green from Applied Biosystems (Warrington, UK) and Roche Diagnostics (Mannheim, Germany). TaqMan reverse transcription kit reagents MicroAmp Optical Reaction Plate and High-Capacity cDNA revers transcription kit were from Applied Biosystems (Warrington, UK), Agilent Total RNA isolation kit from Agilent Technologies (Santa Clara, CA, US) and TRIzol Reagent from Invitrogen Dynal AS (Carlsbad CA, US). Immun-Star WesternC kit, Mini-Protean TGX gels were from BioRad (Copenhagen, Denmark). SPE column was from Macherey-Nagel (Düren, Germany).

### Donor characteristics

Muscle biopsies were obtained from subjects undergoing bariatric surgery at The Morbid Obesity Center at Vestfold Hospital Trust, Norway. Biopsies were obtained after informed written consent and approval by the Regional Committee for Medical and Health Research Ethics, Oslo, Norway (approvement S-09078d). Blood samples were taken the day before biopsy retrieval or earlier (range 1 month to 2 years, median 1.5 years), due to practical reasons. The diagnosis of type 2 diabetes was based on fasting plasma glucose ≥7.0 mM, HbA_1c_ ≥ 6.5% and/or the use of one or more antidiabetic drug. Of the 14 donors in the type 2 diabetic group, 6 were on metformin monotherapy, 2 on metformin and glimepiride, 1 on metformin in combination with rosiglitazone, 1 on pioglitazone monotherapy, 2 on insulin and 2 were untreated. The donors did not receive medication on the day of surgery.

### Cell culturing

Satellite cells were isolated from *M*. *obliquus internus abdominis* and cultured as previously described [28]. Experiments were performed after 7–8 days of differentiation and 100 μM OA was added the last 24h of differentiation period. Protein concentration in each sample was determined [29], and the results were standardized according to this value for each well. Not all donors were included in each set of experiment, but 5–8 donors in each group were used, and these were carefully matched with respect to age and BMI.

### Deoxyglucose uptake

Myotubes were preincubated for 1h in serum-free DMEM-Glutamax (5.5 mM glucose) ± 100 nM insulin at 37°C before addition of [^3^H]deoxyglucose (37 kBq, 0.1 μM) in presence or absence of 100 nM insulin. Deoxyglucose uptake was measured for 1 h as previously described [30].

### Scintillation proximity assay

Scintillation proximity assay (SPA) was performed as previously described [31] with [1–^14^C]oleic acid (OA) (18.5 kBq, 100 μM) in medium without phenol red. Briefly, [^14^C]OA taken up and accumulated by adherent cells will be concentrated close to the scintillator embedded in the plastic bottom of each well (Scintiplate, Perkin Elmer) and provide a stronger signal than the dissolved in the medium alone [32]. Lipid accumulation was monitored up to 24h with liquid scintillation. Thereafter, the cells were washed twice with DPBS with 0.5% BSA, and incubated in DPBS without radioactivity and liquid scintillation measurements were monitored up to 3h. The decline in [^14^C]OA present in the cells in presence of triacsin C (total lipolysis, 10 μM) [33] was determined, before the remaining cell-associated (CA) radioactivity was assessed. The decline in [^14^C]OA represent radioactivity released by the cells and provide a measure of lipolysis. Triacsin C inhibits long-chain fatty acyl-CoA synthetase and will therefore inhibit, among other pathways, re-esterification.

### Lipid distribution

Myotubes were incubated with 100 μM OA (18.5 kBq, 100 μM) for 24 h. Myotubes were then washed twice with PBS and harvested with two additions of 125 μl distilled water. Cellular lipids were extracted as described earlier [5]. Briefly, homogenized cell fractions were extracted, lipids were separated by thin layer chromatography, and radioactivity was quantified by liquid scintillation. The amount of neutral lipids was related to total protein concentrations.

### Determination of total TAG content

Total TAG content in the cells were measured as previously described [34]. Briefly, lipids were extracted in dichloromethane:methanol:water (2.5:2.5:2.1 (v/v/v)) in presence of internal standards. Neutral lipids were separated over a SPE column (glass Chromabond pure silica, 200 mg), and neutral lipids were eluted with chloroform:methanol (9:1 (v/v)). The organic phase was evaporated to dryness and dissolved in 20 μl of ethyl acetate. 1 μl of the lipid extract was analyzed by gas-liquid chromatography.

### TAG hydrolase activity assay

TAG hydrolase activity was measured on cell lysates as previously described [35]. [9,10-^3^H]triolein was emulsified with phospholipids by sonication and used to determine TAG hydrolase activity [5].

### Live imaging

Myotubes were cultured on 6-well glass bottom plates coated with ECM gel. The cells were incubated with Hoechst 33258 to stain nuclei, Bodipy 493/503 to stain neutral lipids and LDs and MitoTrackerRed FM to stain mitochondria as previously described [5]. Images were randomly taken in 25–36 positions per well. After gating out aggregates and dead cells, each parameter was determined from about 240 images per donor group (average of 38±4 nuclei per image).

### Substrate oxidation assay

Myotubes were cultured on 96-well CellBIND microplates. Substrates, [1–^14^C]OA (18.5 or 37 kBq, 5 or 100 μM) or D-[^14^C(U)]glucose (37 or 21.5 kBq, 111 or 200 μM/l) were given during 4h CO_2_ trapping with or without 5 mM glucose or 100 μM/l OA present. A 96-well UniFilter-96 GF/B microplate was mounted on top of the CellBIND plate and CO_2_ production was measured in DPBS medium with 10 mM HEPES and 1 mM L-carnitine for 4h, as previously described [32]. The sum of ^14^CO_2_ and remaining CA radioactivity reflects total cell uptake. Incomplete fatty acid oxidation, assessed as acid soluble metabolites (ASMs), was measured as described [31].

### Metabolic parameters

Suppresibility is the ability of the cells to decrease OA oxidation by acutely added glucose, adaptability is the ability to increase OA oxidation with increasing OA concentration and substrate-regulated flexibility is the ability to increase OA oxidation while changing from “fed” (low fatty acid, high glucose) to “fasted” (high fatty acid, no glucose added) condition [5].

### RNA isolation and mRNA expression

Total RNA from cells was isolated by Agilent Total RNA isolation kit according to the supplier’s protocol. Total RNA from muscle biopsies was isolated using TRIzol and a clean-up procedure was performed using RNeasy kit. RNA was reverse-transcribed with oligo primers using a heat block (25°C for 10 min, 37°C for 1 h and 99°C for 5 min) or a PerkinElmer Thermal Cycler 9600 and qPCR was performed using an ABI PRISMT 7000 Detection System or a LightCycler 480 (Roche Diagnostic, Mannheim, Germany). Forward and reverse primers used at concentration of 30 μM are presented in S1 Table. The transcription levels were normalized to the average of housekeeping genes *GAPDH* and *RPLP0*.

### Immunoblotting

Total cell lysates prepared either in Laemmli or RIPA buffer containing 10 μl/ml protease inhibitor, 10 μl/ml phosphatase I inhibitor and 10 μl/ml phosphatase II inhibitor were electrophoretically separated, blotted to nitrocellulose membrane and incubated with antibodies recognizing human total and phosphorylated Akt (Ser473), total HSL (#4107), Ser660 HSL (#4126) and ATGL (#2138, all from Cell Signaling Technology, Beverley, MA, US), comparative gene identification 58 (CGI-58) (#H00051099-M01, Abnova, Tapei, China), PLIN2 (#PA5-25042) and PLIN3 (#PA5-20272, Thermo Scientific, France), myosin slow muscle fiber (MAB1628, Millipore Billerica, MA, US), total OXPHOS WB antibody cocktail (#110411, Abcam), alpha-tubulin rabbit (#2144), GAPDH (#5174) and β-actin (#4970, all from Cell Signaling Technology). Immunoreactive bands were visualized with enhanced chemiluminescence (Chemidoc XRS, BioRad) and quantified with Gel-Pro Analyzer (version 2.0) software. Antibodies against GAPDH, alpha-tubulin or β-actin were used to normalize the protein-antibody signal.

### Presentation of data and statistics

Two-tailed unpaired Student’s t-tests were performed to determine the difference between the donor groups (GraphPad Prism 5.0, GraphPad Software Inc., San Diego, CA, US). For correlation studies, Spearman correlation analysis was performed and Spearman coefficient, ρ, defines the strength of the correlation (GraphPad Prism). Linear mixed model analysis (LMM, SPSS 20.0.0.1, IBM SPSS Inc., Chicago, IL, US) was used to compare the donors in time-course fatty acid accumulation and lipolysis experiments (SPA). Values are reported as means ±SEM if not stated otherwise. The value *n* represents the number of different donors used each with at least duplicate samples. P < 0.05 was considered statistically significant.

## Results

### Cell and donor characteristics

Selected characteristics of the muscle biopsy donors are presented in Table 1. Severely obese without type 2 diabetes and severely obese with type 2 diabetes differed in fasting plasma glucose and in HbA_1c_. There were no differences in plasma insulin, LDL, HDL, cholesterol, or triacylglycerol (data not shown). The severely obese subjects with type 2 diabetes were on average 9 years older than the severely obese subjects with normal glucose tolerance. However, the difference in age could not explain the metabolic differences between the groups, assessed by multivariate analysis, and the duration of obesity in the two groups was not statistically different. Physical activity level (self-reported) was also similar in the two groups (data not shown). Myotube cultures established from the two groups of donors revealed no difference in protein or mRNA expression of the marker for muscle fiber type I (MYH7, slow fiber type), determined by immunoblotting and qPCR. There were no differences in mRNA expression of myosin heavy chains in the biopsy samples either (data not shown).

**Table 1:** Donor characteristics for the donor group severely obese non-diabetics (nD) and severely obese with type 2 diabetes (T2D).

	Age (yrs)	BMI (kg/m^2^)	Fasting Glucose (mmol/l)	HbA_1c_ (%)	Insulin (pmol/l)	LDL (mmol/l)	HDL (mmol/l)	Cholesterol (mmol/l)	TG (mmol/l)	*n*	Male
nD	40 ± 7	44 ± 7	5.0 ± 0.5	5.4 ± 0.5	97 ± 51	2.8 ± 0.6	1.2 ± 0.2	4.7 ± 0.8	1.6 ± 0.5	15	4
T2D	49^a^ ± 10	43 ± 6	7.6^a^ ± 1.5	7.1^a^ ± 1.6	78 ± 48	2.4 ± 1.0	1.1 ± 0.3	4.4 ± 1.3	1.8 ± 0.7	14	5

Mean ± SD are presented. ^a^Significantly different from nD. BMI; body mass index, HbA_1c_; glycosylated hemoglobin, HDL; high-density lipoprotein, LDL; low-density lipoprotein, *n*; number, TG; triacylglycerol.

### Myotubes *in vitro* maintain their *in vivo* phenotype

**Fig 1:**
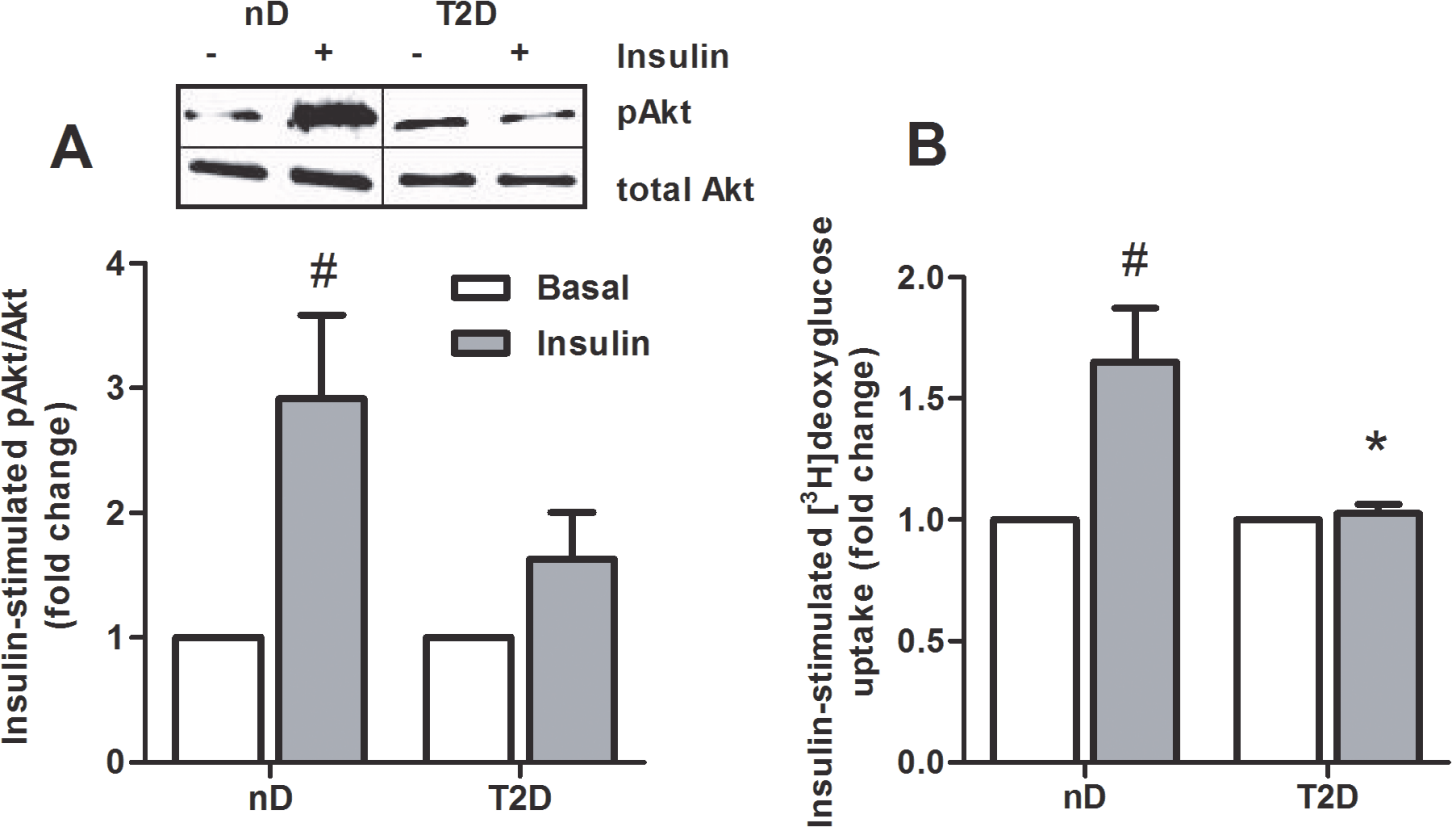
Decreased insulin-stimulated glucose uptake and Akt phosphorylation in myotubes from severely obese donors with type 2 diabetes. (***A***) Myotubes from severely obese non-diabetic donors (nD) and severely obese donors with type 2 diabetes (T2D) were incubated for 15 min with or without 100 nM insulin, before immunoblotting analysis with antibodies against phosho-Akt (Ser473) and total-Akt were performed. Data are shown as ratio phosho-Akt/total Akt and related to unstimulated cells (*n* = 4–5). Immunoblotting from one representative experiment. (*B*) Glucose uptake was measured by [^3^H]deoxyglucose with or without 100 nM insulin for 1 h. Basal glucose uptake was 251 ± 68 nmol/mg protein (nD) and 203 ± 34 nmol/mg protein (T2D). Data are presented as mean ± SEM normalized to unstimulated cells, *n* = 7–8, ^#^p < 0.05 versus basal, *p < 0.05 versus nD.

To confirm that the myotubes maintained their diabetic phenotype in culture, insulin-stimulated glucose uptake and Akt phosphorylation (Ser473) were measured. Insulin-stimulated phosphorylation of Akt tended to be lower in myotubes from severely obese donors with type 2 diabetes compared to severely obese donors with normal glucose tolerance (Fig. 1A, p = 0.11). Insulin-stimulated glucose uptake was abolished in myotubes from type 2 diabetics compared to cells from non-diabetics (Fig. 1B, p = 0.01), implying a conserved insulin resistance in cultured cells.

### Reduced lipid accumulation in type 2 diabetic myotubes

**Fig 2:**
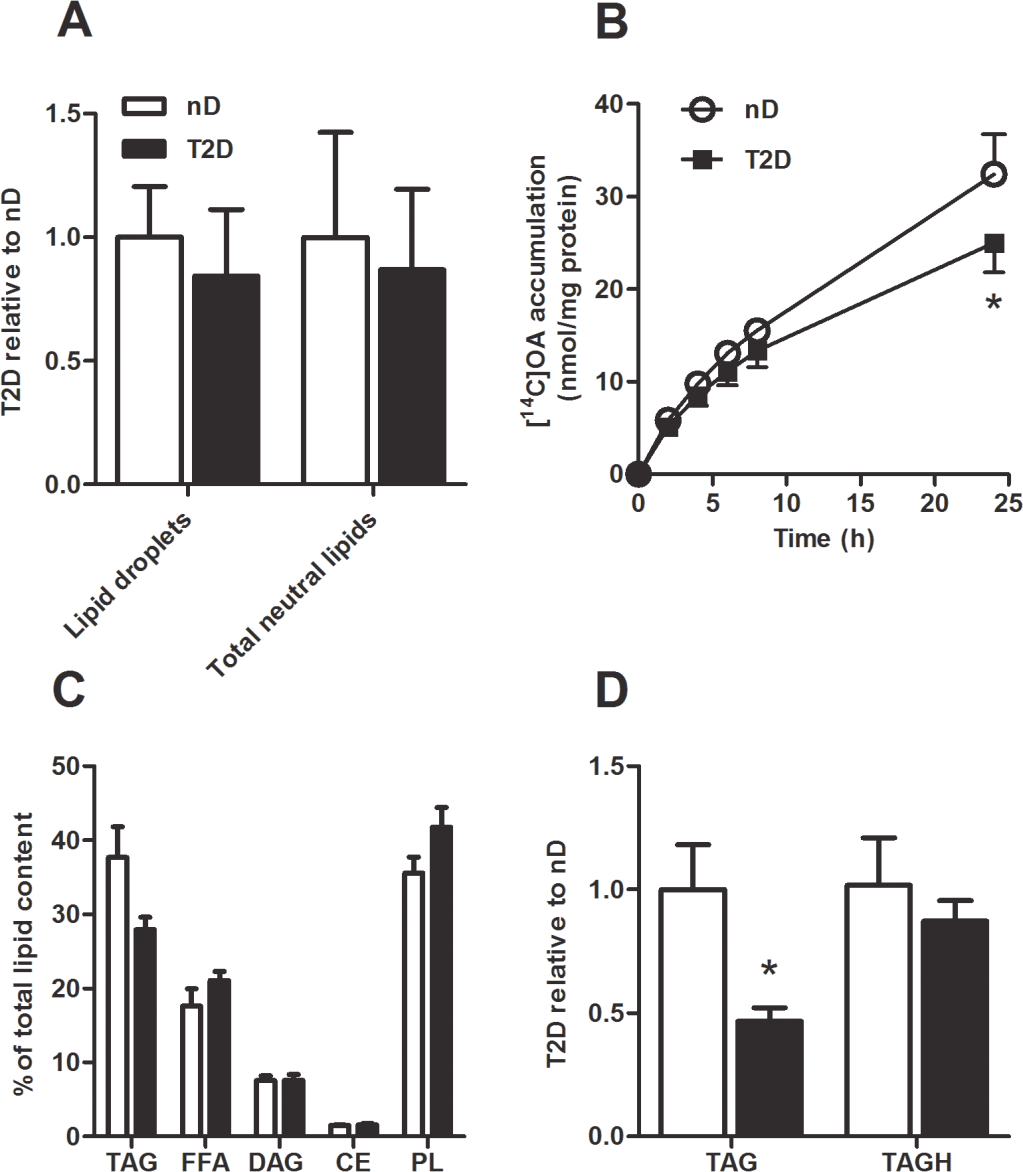
Lower lipid accumulation in myotubes from severely obese donors with type 2 diabetes. (*A*) Live imaging of lipid droplets and total neutral lipid content in myotubes from severely obese non-diabetic donors (nD) and severely obese donors with type 2 diabetes (T2D). The cells were incubated for 15 min with Hoechst 33258 to stain nuclei and Bodipy 493/503 to stain neutral lipids, *n* = 5. (*B*) [^14^C]OA accumulation over 0–24 h. *n* = 5–6. (*C*) Lipid distribution measured as incorporation of [^14^C]OA into TAG, FFA, DAG, CE and PL. Data are presented as % of total lipids in the cell, *n* = 5. (*D*) Total cell content of TAG and TAG hydrolase (TAGH) activity in non-diabetic and type 2 diabetic myotubes after 24 h incubation with 100 μM OA. Total cell content of TAG was 1.4±0.2 nmol/mg protein (T2D) and 3.0±0.6 nmol/mg protein (nD). TAGH was 3.7±0.3 nmol mg protein^−1^ h^−1^ (T2D) and 4.1±0.8 nmol mg protein^−1^ h^−1^ (nD) Data are presented relative to mean values of non-diabetics, *n* = 6–7. *p<0.05 versus non-diabetic. CE, cholesteryl ester; DAG, diacylglycerol; FFA, free fatty acid; OA, oleic acid; PL, phospholipid; TAG, triacylglycerol.

To evaluate lipid turnover in the cells, accumulation of oleic acid (OA), distribution of lipids and TAG hydrolase activity were determined. Without pre-incubation with OA, there were no differences in lipid droplet number (LD) or total neutral lipid content in myotubes from severely obese donors with or without T2D (Fig. 2A, p = 0.65, p = 0.81). Cellular accumulation of [^14^C]OA was significantly lower in myotubes from type 2 diabetic subjects than in myotubes from non-diabetics (Fig. 2B, p = 0.035). TAG measured after incubation of [^14^C]OA for 24 h tended to be lower in myotubes from type 2 diabetic subjects than in myotubes from non-diabetics (Fig. 2C, p = 0.058) with no differences in free fatty acid (FFA, p = 0.22), diacylglycerol (DAG, p = 0.97), cholesteryl ester (CE, p = 0.59) or phospholipids (PL, p = 0.10). In line with this, total cell content of TAG measured after incubation of OA for 24 h was 50% lower in myotubes from type 2 diabetic subjects than in myotubes from non-diabetics (Fig. 2D, p = 0.01). Although there was no difference in TAG hydrolase (TAGH) activity (Fig. 2D, p = 0.48), the ratio DAG/TAG tended to be higher in the myotubes from type 2 diabetic subjects (27%, p = 0.086).

### Higher lipolysis rate in type 2 diabetic myotubes

**Fig 3:**
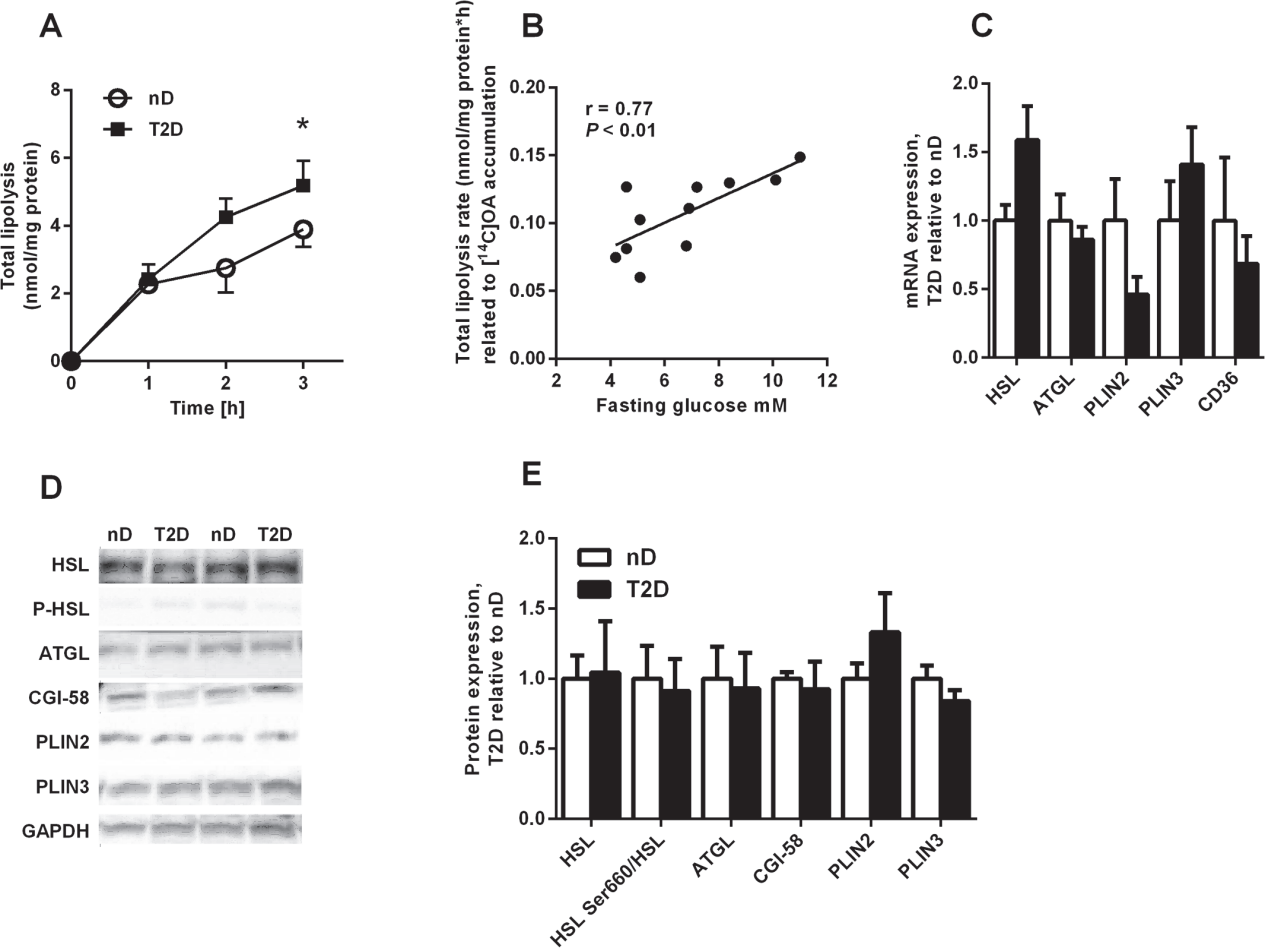
Higher lipolysis rate in myotubes from severely obese donors with type 2 diabetes. (*A*) Total lipolysis (with triacsin C) after 24 h incubation with [^14^C]OA in non-diabetic (nD) and type 2 diabetic myotubes (T2D) *n* = 5–6. (*B*) Fasting plasma glucose levels correlated positively with total lipolysis rate, *n* = 11 (C) mRNA expression of hormone-sensitive lipase (HSL), adipose triglyceride lipase (ATGL), perilipin 2 (PLIN2), perilipin 3 (PLIN3) and the fatty acid transporter CD36. Data are presented relative to mean values of non-diabetics, *n* = 6–10. (*D-E*) Protein expression of HSL, ATGL, comparative gene identification 58 (CGI-58), PLIN2 and PLIN3 and phosphorylation of HSL at serine 660 (HSL Ser660) after 24h incubation with 100 μM OA. (D) Two representative blots are shown. Data are presented relative to mean values of non-diabetics, *n* = 5.

To evaluate whether increased lipolysis rate could explain the lower accumulation and cell content of TAG in type 2 diabetic myotubes, lipolysis rate with triacsin C (total lipolysis) was measured. Indeed, lipolysis in presence of triacsin C was significantly higher in type 2 diabetic myotubes than in non-diabetic myotubes (Fig. 3A, p = 0.046) and total lipolysis rate related to cell-associated OA correlated positively with fasting plasma glucose levels of the subjects (Fig. 3B). However, there were no differences in mRNA or protein expression of the lipases HSL and ATGL and the lipase cofactor CGI-58, or the lipid droplet proteins PLIN2 and PLIN3 between the two groups (Fig. 3C, D). The fatty acid transporter CD36 was also equally expressed (Fig. 3C). These genes were also measured in presence of OA, but this revealed no further differences between the groups (data not shown). No differences were observed in biopsy samples either (S2 Table). Phosphorylation of HSL at serine 660, which is associated with activation of the lipase, was neither different between myotubes from the two groups (Fig. 3D).

### Reduced mitochondrial staining but unchanged substrate oxidation

**Fig 4:**
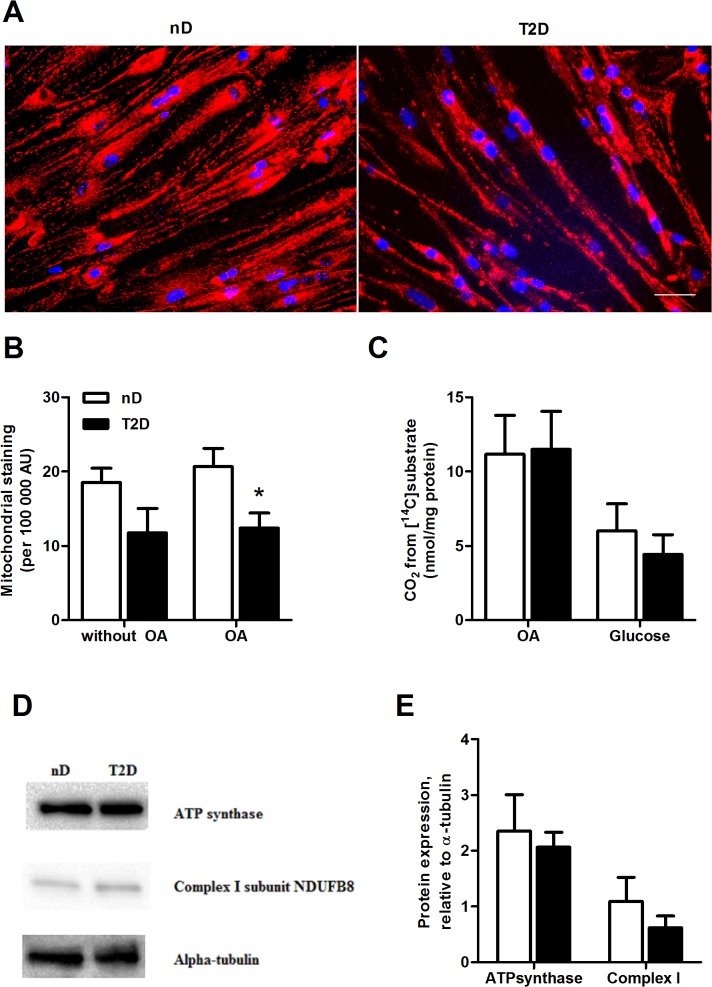
Lower mitochondrial staining of myotubes from severely obese donors with type 2 diabetes. Myotubes from severely obese non-diabetic donors (nD) and severely obese donors with type 2 diabetes (T2D) were stained with MitoTrackerRed (mitochondria) and Hoechst 33258 (nuclei). (*A*) Representative images after 24h incubation with 100 μM oleic acid (OA). Blue is nuclei and red is mitochondria. Magnification is 1:20, scale bar represents 50 μm. (*B*) Mitochondrial staining related to number of nuclei per 100 0000 AU. Myotubes were incubated with or without 100 μM oleic acid (OA). Mean±SEM are presented related to number of nuclei, *n* = 5, *p<0.05 versus non-diabetics. *(C)* OA oxidation or glucose oxidation were measured in presence of [^14^C]OA (18.5 kBq, 100 μM) or [^14^C]glucose (18.5 kBq, 200 μM) for 4h. Data are presented as mean±SEM, *n* = 7. AU, arbitrary units. *(D-E)* Protein expressions of ATP synthase subunit and Complex I subunit NDUFB8. Protein samples were harvested and analyzed as described in Materials and Methods, and expression levels were normalized to alpha-tubulin. *(D)* Representative Western blots from one experiment. *(E)* Protein expressions of ATP synthase subunit and Complex I subunit NDUFB8 related to alpha-tubulin. Data are presented as mean±SEM, *n* = 5–8.

About 40% lower mitochondrial staining, measured as MitoTrackerRed intensity, was observed in type 2 diabetic compared to non-diabetic myotubes (Fig. 4A, B, p = 0.03). Glucose and OA oxidation (Fig. 4C, p = 0.5, p = 0.93, respectively) were not statistically different between the groups, neither were OA and glucose uptake nor incomplete OA oxidation (ASMs) (S1 Fig.). Further, expressions of the two OXPHOS proteins, ATP synthase unit and Complex I subunit NDUFB8, did not differ significantly between the groups either (p = 0.058 and p = 0.14, respectively, Fig. 4D, E). We were not able to detect Complex II subunit 30 kDa, Complex III subunit Core 2 and Complex IV subunit 2 of the OXPHOS protein complex. Challenging the cells with the mitochondrial uncoupler FCCP (carbonyl cyanide 4-trifluoromethoxy phenylhydrazone, 1 μM) or higher OA concentrations (600 μM) revealed no further difference between groups (data not shown). Moreover, there were no differences between the groups in mRNA expression of essential genes in energy metabolism, e.g. carnitine palmitoyltransferase 1B (*CPT1B*), pyruvate dehydrogenase kinase isozyme 4 (*PDK4*), peroxisome proliferator-activated receptor gamma coactivator-1 alpha *(PPARGC1A*) and cytochrome C-1 (*CYC1*) (data not shown).

### Metabolic flexibility

**Fig 5:**
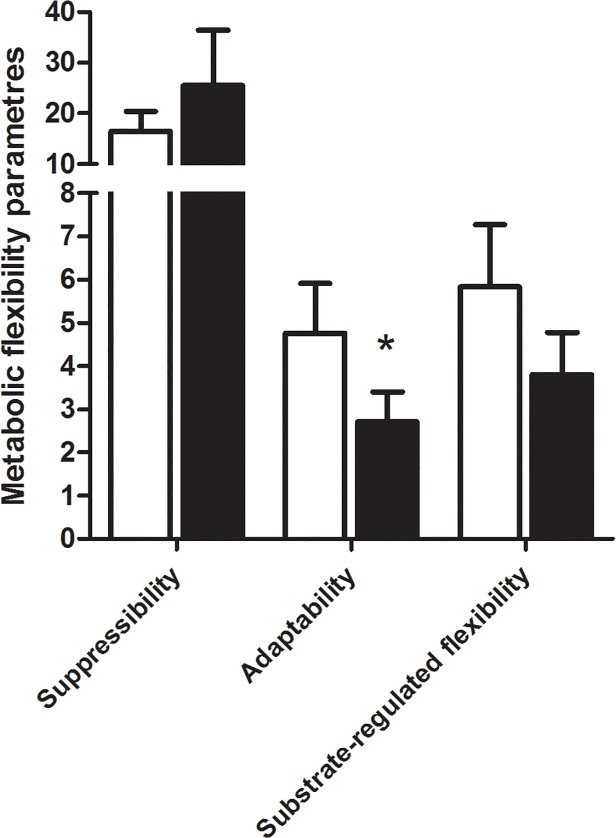
Metabolic flexibility parameters in myotubes derived from severely obese non-diabetic donors (nD) and severely obese donors with type 2 diabetes (T2D). Calculations of metabolic parameters are performed as described [5]. Suppressibility is (1- oxidation of 100 μM OA at 5 mM glucose/oxidation of 100 μM OA at no glucose added)*100%, adaptability is oxidation of 100 μM OA/oxidation of 5 μM OA, and substrate-regulated flexibility is oxidation of 100 μM OA without glucose added/oxidation of 5 μM OA at 5 mM glucose. Linear mixed model was performed for adaptability and suppressibility, while unpaired two-tailed t-test was performed for substrate-regulated flexibility, *p<0.05 versus non-diabetics, *n* = 7–8.

The metabolic flexibility parameters were determined as described before [5], and myotubes derived from type 2 diabetics showed an about 30% lower adaptability for OA oxidation than myotubes from non-diabetics, whilst the substrate-regulated flexibility and suppressibility parameters were not significantly different (Fig. 5, p = 0.02, p = 0.26, p = 0.45, respectively).

## Discussion

Disturbances in skeletal muscle lipid metabolism are clearly associated with insulin resistance and type 2 diabetes. In the present study, we wanted to examine lipid handling in myotubes isolated from severely obese that have developed type 2 diabetes and severely obese with normal glucose tolerance to see whether there are fundamental differences. We observed that myotubes established from severely obese type 2 diabetic donors had higher lipolysis rate and were less able to accumulate fatty acids than myotubes from severely obese with normal glucose tolerance. In line with this, TAG content was lower in myotubes from type 2 diabetic donors but there were no significant differences in lipid distribution into FFA, DAG, CE or PL. There were no differences in fatty acid and glucose oxidation either. Although the diabetic cells had lower mitochondrial content as assessed by Mitotracker, expression levels of the OXPHOS proteins did not differ significantly between the groups. Further, diabetic cells showed less ability to increase fatty acid oxidation with increased oleic acid availability (adaptability). We also confirmed that myotubes derived from severely obese donors with T2D had lower insulin sensitivity compared to cells from non-diabetic donors, a trait apparently conserved from the *in vivo* situation.

There are numerous studies showing an association between insulin resistance and increased lipid accumulation in skeletal muscle of type 2 diabetic subjects [25, 36]. However, there are also indications that this association is not solely due to ectopic lipid accumulation, but rather caused by dysregulation of lipolysis and/or lipid turnover [25, 27, 37]. Lower lipid accumulation and higher lipolysis without correspondingly increased fatty acid oxidation, as observed in type 2 diabetic myotubes in this study, could contribute to accumulation of lipotoxic intermediates, which are reported to interfere with insulin signaling [38–40], (reviewed in [25]). Although we could not observe any differences in DAG content, the ratio of DAG/TAG tended to be higher in type 2 diabetic myotubes. In correspondence with this, higher DAG level in muscle has been associated with obesity and insulin resistance in both rats and humans [39, 41], while muscle ceramides are also elevated in lean or obese insulin resistant humans and rats [41]. Although TLC is a sensitive method, we cannot exclude that small differences that we were not able to detect, in for instance DAG or other lipid intermediates could mediate important signaling effects.

We observed lower lipid accumulation and higher lipolysis rate in myotubes from severely obese type 2 diabetic donors without any difference between cells from the two groups with respect to protein and mRNA expression levels of CD36, PLINs, ATGL, HSL and CGI-58. Reduced protein expression of the lipase HSL has previously been shown in obese insulin-resistant subjects [24, 42], and increased protein levels of ATGL in both obese subjects with type 2 diabetes [22] and obese non-diabetic subjects [24]. However, no apparent changes in lipases and PLIN proteins were observed in the present study despite functional changes in lipolysis. The regulation of lipase activity is a complex process regulated at multiple steps, including phosphorylation of lipases and PLIN proteins and complex movement of these different partners between the lipid droplets and the cytosol [43]. HSL activity is probably mostly regulated by phosphorylation [43]. However, we were not able to detect any differences in HSL660 phosphorylation in myotubes from the two donor groups, but other phosphorylation sites, not studied here, may be involved. Recently, we observed that increased expression of ATGL by palmitic acid in myotubes, did not increase lipolysis, implying that content and activity are not directly linked [31]. ATGL can also be phosphorylated, although its activity appears mainly controlled by the co-activator CGI-58 [43]. Exactly how lipolysis rate is regulation by PLINs is not known, but PLIN2 has been found co-localized with HSL after epinephrine stimulation, and PLIN5 is suggested to channel fatty acids to the mitochondria after lipolysis [43]. In accordance with a recent observation in myotubes from obese individuals with or without type 2 diabetes [44], we could not detect any change in expressions of PLIN2 and PLIN3, and further investigation on how PLINs and lipases interact is needed.

TAG content of type 2 diabetic myotubes was about 50% of the content in non-diabetic controls after 24 h incubation with OA. This difference can possibly be explained by increased lipolytic rate, combined with less lipid accumulation. However, there might also be differences in TAG synthesis, for instance DGAT activity or in lipid uptake. A complete picture is difficult to interpret. CD36 expression was similar as well as fatty acid uptake, assessed as cell associated OA after 4 h (data not shown), and Sparks et al. showed very recently that DGAT-activity did not differ between myotubes from obese males with T2D and BMI and age matched controls [44]. Altogether, we have documented disturbed lipid handling, but still the explanation is puzzling.

In addition to lipid turnover, metabolic flexibility was also impaired in myotubes derived from type 2 diabetic donors, assessed as reduced adaptability for fatty acid oxidation. In support, lower ability to increase oxidation with increasing fatty acid concentration in myotubes from overweight/obese type 2 diabetics [8] and reduced metabolic flexibility *in vivo* in insulin resistant muscle [4] compared to healthy lean donors have been reported. The combination of decreased adaptability and higher lipolysis rate might be unfavorable and likely contribute to an abnormal intracellular lipid profile. Interestingly, we found lower mitochondrial staining in myotubes from severely obese with type 2 diabetes compared to cells from severely obese non-diabetic donors, in accordance with earlier studies showing lower mitochondrial content in insulin resistant muscle [17, 45]. This occurred despite no differences in fatty acid oxidation, nor in the OXPHOS protein expression or mRNA expression of *CYC1*, *PPARGC1A*, *CPT1B* or *PDK4* in myotubes from severely obese donors with type 2 diabetes compared to cells from severely obese non-diabetic donors. The focus in recent years has been directed towards a mitochondrial deficiency/dysfunction in relation to development of type 2 diabetes, and several studies conclude that the observed reduced mitochondrial function in type 2 diabetes is due to, and secondary to, a lower mitochondrial content in muscle (reviewed in [46]). Although one might expect that oxidation and mitochondrial content are positively associated, an inverse correlation between mitochondrial content and degree of complete lipid oxidation has been observed in myotubes [47].

Increasing age is related to elevated insulin resistance and so is increasing obesity related to aging. The subjects with type 2 diabetes in this study were in average older than the non-diabetic subjects, however, the duration of obesity did not differ and age *per se* could not explain the metabolic differences. It is also hypothesized that obesity and inactivity rather than age *per se* is the primary determinant of age-related declines in insulin sensitivity [48, 49].

Altogether, our results show that there are retained differences in cells cultured from subjects with and without diabetes. This is in accordance with many previous observations [6, 14, 44]. Actually, how these differences are conserved from the *in vivo* to the *in vitro* situation is not known. Most studies have failed to explain this by genetic polymorphism, but a possibility, yet undetected, is that epigenetic changes could accumulate *in vivo* and be transmitted to in the cultured cells, causing a diabetic phenotype.

In conclusion, myotubes established from severely obese subjects with established type 2 diabetes had lower ability for lipid accumulation and higher lipolysis rate than myotubes from severely obese donors without diabetes. Our hypothesis is that by storing more lipids as triacylglycerol, the formation of lipotoxic intermediates that could interfere with insulin signaling may be prevented. Higher lipolysis not followed by a higher fatty acid oxidation, in combination with lower adaptability and lower mitochondrial content could contribute to a diabetic metabolic profile.

## Supporting Information

S1 Fig Oleic acid and glucose uptake and acid soluble metabolites (ASM) in myotubes derived from severely obese non-diabetics (nD) and severely obese diabetics (T2D). (*A*) Cellular uptake of oleic acid and glucose, assessed as the sum of cell associated and CO_2_-trapped radioactivity after 4 h, *n* = 7. (*B*) Incomplete fatty acid oxidation, detected as ASM, were assessed after incubation of 100 μM or 600 μM oleic acid (OA) for 24 h, *n* = 5. Data are presented as mean ± SEM.
(TIF)

S1 Table Primer sequences used in real time RT-PCR. (PDF)

S2 Table mRNA expression in biopsies for the donor groups severely obese non-diabetics (nD) and severely obese with type 2 diabetes (T2D). Data are presented as fold change to the average of two housekeeping genes (*GAPDH* and *RPLP0*). Mean ± SEM are presented for *n* = 7 donors per group.
(PDF)
