# Supplementary figures and images for: Myotubes from Severely Obese Type 2 Diabetic Subjects Accumulate Less Lipids and Show Higher Lipolytic Rate than Myotubes from Severely Obese Non-Diabetic Subjects
Source: PLoS One. 2015 Mar 19;10(3):e0119556. doi: 10.1371/journal.pone.0119556 (PMC4366103; doi:10.1371/journal.pone.0119556)

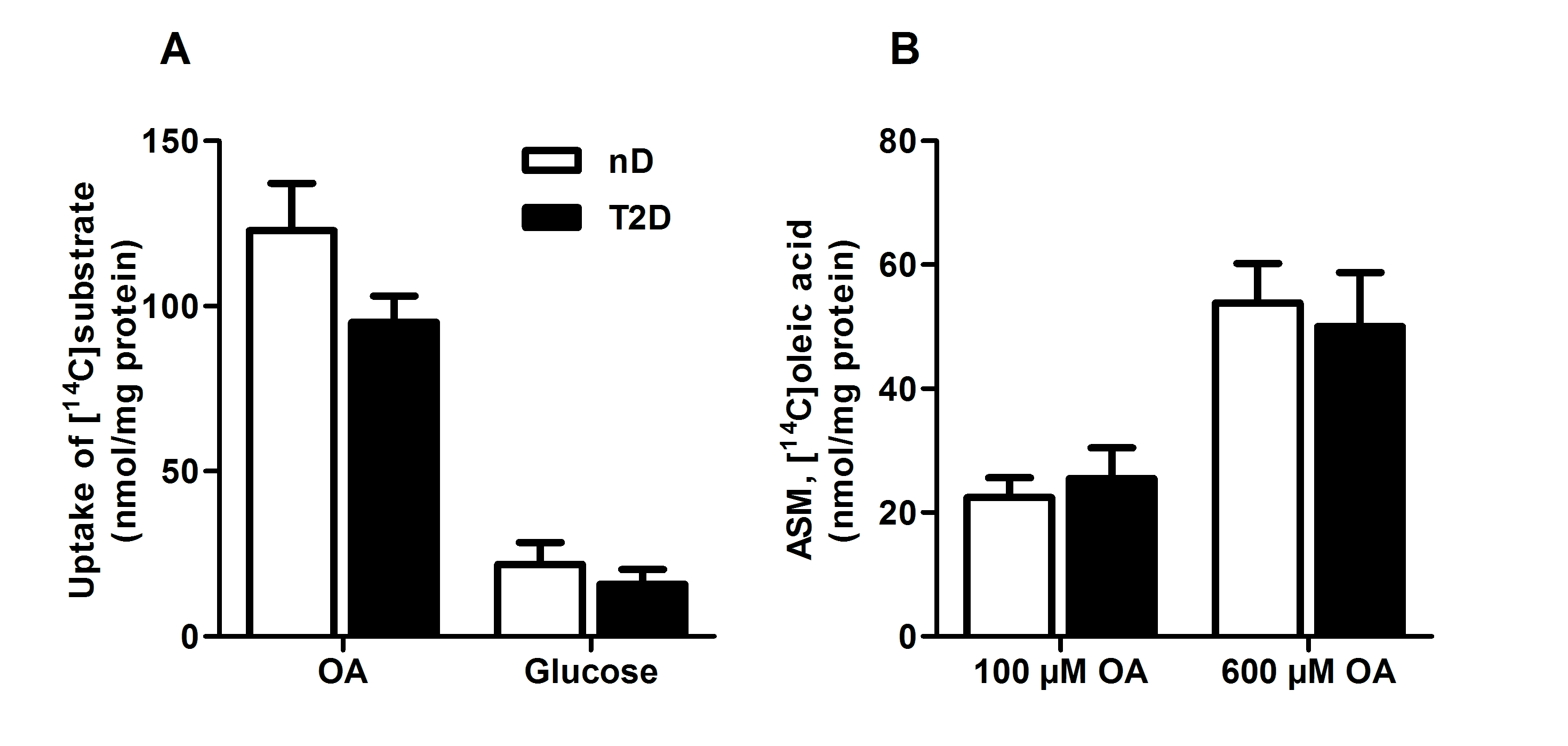

Supplement: S1 Fig — (A) Cellular uptake of oleic acid and glucose, assessed as the sum of cell associated and CO2-trapped radioactivity after 4 h, n = 7. (B) Incomplete fatty acid oxidation, detected as ASM, were assessed after incubation of 100 μM or 600 μM oleic acid (OA) for 24 h, n = 5. Data are presented as mean ± SEM. (TIF) [file pone.0119556.s001.tif]
