# Supplementary material for: Myotubes from Severely Obese Type 2 Diabetic Subjects Accumulate Less Lipids and Show Higher Lipolytic Rate than Myotubes from Severely Obese Non-Diabetic Subjects
Source: PLoS One. 2015 Mar 19;10(3):e0119556. doi: 10.1371/journal.pone.0119556 (PMC4366103; doi:10.1371/journal.pone.0119556)
Supplement: S1 Table — (PDF) [file pone.0119556.s002.pdf]

Table S1. Primer sequences.

Gene	acc_no	Forward	Reverse
RPLP0	M17885	CCATTCTATCATCAACGGGTACAA	AGCAAGTGGGAAGGTGTAATCC
GAPDH	NM002046	TGCACCACCAACTGCTTAGC	GGCATGGACTGTGGTCATGAG
CD36	L06850	AGTCACTGCGACATGATTAATGGT	CTGCAATACCTGGCTTTTCTCAA
PLIN2	NM001122	GGTGATGGCAGGCGCAT	TAGAAGTGAGGAGGCTGTCAGACA
PLIN3	NM001164189.1	CACTCGCTGGGCAAGCTT	CTTGACAGTTTCCATCAGGCTTAG
ATGL	BC017280	TCAGACGGCGAGAATGTCATTAT	TGC AGACATTGGCCTGGAT
HSL	NM005357	ACCTGCGCACAATGACACA	TGGCTCGAGAAGAAGGCTATG
PDK4	BC040239	TTTCCAGACCAACCAATTCACA	TGCCCGCATTGCATTCTTA
CPT1B	L39211	CGGTGGAACAGAGGCTGAA	CGAGGCGATACATATGCTGATG
PPARGC1A	NM013261.3	AAAGGATGCGCTCTCGTTCA	TCTACTGCCTGGAGACCTTGATC
CYC1	NM001916	CTGCCAACAACGGAGCATT	CGTGAGCAGGGAGAAGACGTA
MYH7	NM000257.2	CTCTGCACAGGGAAAATCTGAA	CCCCTGGAGACTTTGTCTCATT
MYH1	NM005963	CCAGACTGTGTCTGCTCTCTTCAG	CAGGACAAGCTCATGCTCCAT
MYH2	NM017534	AAGGTCGGCAATGAGTATGTCA	CAACCATCCACAGGAACATCTTC
