# Supplementary material for: Myotubes from Severely Obese Type 2 Diabetic Subjects Accumulate Less Lipids and Show Higher Lipolytic Rate than Myotubes from Severely Obese Non-Diabetic Subjects
Source: PLoS One. 2015 Mar 19;10(3):e0119556. doi: 10.1371/journal.pone.0119556 (PMC4366103; doi:10.1371/journal.pone.0119556)
Supplement: S2 Table — Data are presented as fold change to the average of two housekeeping genes (GAPDH and RPLP0). Mean ± SEM are presented for n = 7 donors per group. (PDF) [file pone.0119556.s003.pdf]

**Table S2. mRNA expression in biopsies for the donor groups severely obese non-diabetics (nD) and severely obese with type 2 diabetes (T2D).**

| | CD36 | PLIN2 | PLIN3 | ATGL | PDK4 | CPT1B |
|---|---|---|---|---|---|---|
| nD | 2.43±0.28 | 1.62±0.15 | 0.37±0.04 | 1.76±0.10 | 2.21±0.50 | 2.40±0.30 |
| T2D | 2.70±0.63 | 1.63±0.28 | 0.48±0.05 | 1.90±0.27 | 2.43±0.42 | 2.59±0.31 |

Data are presented as fold change to the average of two housekeeping genes (*GAPDH* and *RPLP0*). Mean ± SEM are presented for *n* =7 donors per group.
